# What Is Your Faction? Multidimensional Evidence for the Divergent Series As the Basis for a New Model of Personality and Work Life

**DOI:** 10.3389/fpsyg.2017.01751

**Published:** 2017-10-06

**Authors:** Bruno C. de Souza, Antonio Roazzi

**Affiliations:** ^1^Department of Administrative Sciences, Graduate Program in Business Administration, Universidade Federal de Pernambuco, Recife, Brazil; ^2^Departmento of Psychology, Universidade Federal de Pernambuco, Recife, Brazil

**Keywords:** personality dimensions, Facet Theory, work, fiction, Divergent trilogy

## Abstract

**Introduction:** The successful “Divergent” sci-fi trilogy by writer Veronica Roth portrays a dystopian and post-apocalyptic society where the population is divided into five groups called “Factions,” each with a specific social role and associated to a specific set of psychological traits. Though fictional, such typology is compelling and may provide a significant contribution to personality studies.

**Objectives:** To investigate the accuracy of the classification of psychological and sociocultural traits into five Factions as described in Divergent and their potential practical usefulness for understanding work life choices and experiences in organizations.

**Method:** A total of 217 Brazilian adult men and women of various ages, socioeconomic status and ethnicities were submitted to measures of several psychological and sociocultural variables, as well as of how strongly they supposedly manifest each Faction. The resulting dataset was studied using Smallest Space Analysis (SSA) and Facet Theory.

**Results:** The Factions were shown not only to be associated to psychological variables in ways consistent with the descriptions from Divergent, but also to be related to specific aspects of one’s work life in organizations.

**Conclusion:** The five Factions conceived by Roth appear to constitute an original set of constructs that are psychologically valid and, at the same time, of practical use in predicting work life choices and experiences. This justifies engaging in future empirical and theoretical work toward a new scientific model of potential practical value.

## Introduction

Since their origins in the early 20th Century, personality tests were developed to aid organizations in activities such as personnel selection, career counseling, coaching, team formation, evaluation of group dynamics, leadership training, marketing, and management of the quality of life at work (Mischel, 1968; Kaplan and Saccuzzo, 2010). Currently, personality testing for organizational use is a thriving global US$ 2–4 billion dollar industry, in spite of the existence of significant criticism as to its effectiveness and value (The Economist, 2013).

Among the myriad of tests presently utilized in corporations throughout the World, perhaps the most widely used is the Myers Briggs Type Indicator (MBTI), which is also one of the most criticized, particularly in terms of scientific validity and usefulness (Pittenger, 1993a,b; Gardner and Martinko, 1996; Coffield et al., 2004; Morgeson et al., 2007). Also very widely used are the instruments based on the “Big Five” or “OCEAN” personality traits, such as the Revised NEO Personality Inventory (NEO PI-R), the Five-Factor Model Rating Form (FFMRF), and the Big Five Inventory (BFI), which have a much better scientific standing, though it can still be pointed out that they leave something to be desired in terms of theory and practical applications (Hunter et al., 1990; Rosenthal, 1990; Mount and Barrick, 1998; Block, 2010).

Engler (2008) points out that many of the theoretical and even experimental developments in personality studies have come not just from science, but were also originated from a very broad set of concepts and assumptions present in philosophy, religion, and even art. Therefore, future advances in the field may very well arise from ideas inspired by similarly non-scientific sources, including works of pure fiction, with the scientific development and testing of models occurring later. This can be seen as a form of tapping into what Jerome Bruner referred to as the “folk psychology” that emerges in the narratives of a culture (Bruner, 1990).

McCrae et al. (2012) argue that writers of fiction must be able to accurately portray human psychology in order to create good stories. They also demonstrated the existence of characters portrayed in classical literature from Goethe, Voltaire, and Molière that display personalities with psychological coherence, real-life similarity, and even the possibility of a quasi-clinical evaluation according to current personality models.

Crysel et al. (2015) go as far as providing evidence of a fictional writer producing a classification of personality dimensions that seems to mirror at least some elements of scientific personality theories and measures. Especifically, they have shown that the fictional classification of student “houses” described in wildly popular Harry Potter fantasy books seems to have specific and quantifiable associations to different psychometric measures of personality.

Personality types and testing are the central theme in the best-selling trilogy of books written by American fiction writer Veronica Roth, namely, Divergent, Insurgent, and Allegiant (Roth, 2013), which has recently been adapted into a very successful motion picture franchise (The Wrap, 2014). In the series, the fictional dystopian and isolated city of Chicago divides its people into five “Factions,” named Abnegation, Amity, Candor, Dauntless, and Erudite, each of which corresponds to a specific personality type and relates to a specific set of functions in society. The psychological typology presented in this literary work is very compelling, and it is considered as one of the main reasons for its success (Dominus, 2011). The Divergent Series Complete Collection even includes a small, non-validated, test, called “Faction Quiz,” aimed at identifying the reader’s propensity toward each of the Factions (Roth, 2013). There are commentators that have already perceived the Factions as having some analogies to the Big Five personality dimensions (Freeman, 2012) and to the VIA Classification of Character Strengths and Virtues (Niemiec, 2014).

The present paper investigates the scientific validity of the Factions, as described in the Divergent series and measured through the Faction Quiz, in terms of being dimensions that might be associated in specific ways to psychological measures of personality, values, emotional regulation, cognition, and behavior. It also aims to assess how effective such a system might be in terms of a practical use for people management in organizations, particularly predicting an individual’s professional choices and work life experiences.

An early version of this investigation, addressing only the preliminary results regarding the associations between the Factions and psychological variables, has been presented at the 15th International FTA Conference (Souza and Roazzi, 2015). The present paper, on the other hand, includes all the findings, variables, analyses, and interpretations of the study, plus those pertaining to professional choices and work life experiences.

### Human Personality and People Management

The concept of personality can be seen as the very core of Psychology (Atkinson et al., 2000; Santrock, 2008; Feist and Gregory, 2009). Even though there is no universally accepted definition for the term, most authors would agree that it entails a set of individual traits that act upon motivational, emotional, cognitive, and behavioral processes to produce a consistent pattern of thought and action throughout one’s existence, something that involves self-perception, values, and attitudes (Krauskopf and Saunders, 1994). Such traits are also generally defined as being relatively stable in time and exclusive to each person (Feist and Gregory, 2009).

The relevance of the concept of personality for People Management comes from the fact that individual traits can both influence and be influenced by an organization (Paz, 2004). Career counseling, coaching, team formation, evaluation of group dynamics, leadership training, marketing, and management of the quality of life at work are just some of the practical applications of the knowledge on the subject (Mischel, 1968; Kaplan and Saccuzzo, 2010). There are also several studies attempting to associate personality factors to the performance of personnel selection for various careers, as well as to professional success (Santos et al., 2003). Others try to identify the impact of organizational configurations of social power and personality types on the well-being of a company (Dessen and Paz, 2010).

### Personality Theories, Models and Tests

#### The Big Five

The Big Five model of personality traits arose from a study done by Sir Raymond Cattell with 171 adjectives in the English language referring to stable and observable individual traits, from which he built the Sixteen Personality Factor Questionnaire (16PF). By means of multivariate analysis, he discovered five factors that could explain most of the variance in the personality data produced by the 16PF (Tupes and Christal, 1961), a finding later confirmed by Walter Norman (Norman, 1963). In 1980, Lewis Goldberg, Naomi Takemoto-Chock, Andrew Comrey, and John M. Digman revised all the personality measurement instruments available at the time and concluded that the most promising ones were, again, those that contained five factors, similarly to what was previously found by Cattell and Norman (Goldberg, 1980, 1981), which eventually lead to the widespread dissemination and acceptance of the model.

As summed up by Goldberg (1980, 1981) and Digman (1990), the Big Five model identifies five personality factors with the following traits:

• Openness to Experience: Intellectual curiosity, creativity, and a preference for novelty and variety, as well as an appreciation of art;
• Conscientiousness: Self-discipline, emphasis on duty and obligation, aim for achievement, and a preference for planned rather than spontaneous behavior;
• Extraversion: Tendency toward positive emotions, assertiveness, sociability, talkativeness, and search for the company of others, its opposite being Introversion;
• Agreeableness: Inclination to being compassionate, cooperative, trusting, and helpful, having a concern for social harmony and getting along with others;
• Neuroticism: Tendency toward states of anger, fear, and/or depression, vulnerability to anxiety, its opposite being Stability.

The Big Five personality traits have been associated to mental health (Saulsman and Page, 2004), academic achievement (Komarraju et al., 2011), and work success (Hunter et al., 1990; Rosenthal, 1990; Mount and Barrick, 1998), an indication of both the validity and usefulness of the model. However, there is still criticism as to the lack of an actual theory of personality to explain the five factors (Block, 2010), the occurrence of correlations between the traits (van der Linden et al., 2010), the evidence of possible additional traits (Paunonen et al., 2003; Santrock, 2008), and limited applicability for personnel selection (Morgeson et al., 2007).

#### The Myers-Briggs Type Indicator

The Myers Briggs Type Indicator is one of the most widely used in the American and Western European corporate environment (Morgeson et al., 2007; The Economist, 2013). It was originally based on the theory of psychoanalyst Carl Gustav Jung regarding the psychological types, though it later evolved to become a distinct and separate model (Myers and Myers, 1980).

According to Myers and McCaulley (1985), the MBTI assumes that human personality is based on four independent dichotomous functions, namely: Introversion(I)-Extra-version(E), Sensing(S)-Intuition(N), Thinking(T)-Feeling(F), and Judging(J)-Perceiving(P). The basic premise is that people tend to have traits that are situated in one of the two extremes of each function, thereby producing a total of 16 possible combinations (2 × 2 × 2 × 2) that would constitute a fundamental typology for human personality (Myers and Myers, 1980; Myers and McCaulley, 1985). It is also assumed that the four functions follow a hierarchy of dominance, so that a given individual will have one primary function that is the most conscious, secondary and tertiary functions that are intermediate, and a quaternary one that is the most unconscious.

In spite of its immense popularity even within the corporate world, there is strong scientific criticism aimed at the MBTI, particularly regarding its questionable statistical validity, low test-retest reliability, and dubious or biased evidence as to its practical value in professional and organizational contexts (Pittenger, 1993a,b; Gardner and Martinko, 1996; Coffield et al., 2004).

#### Other Influential Models

There are many personality theories, models, and instruments that, even though not as widespread as the MBTI and the Big Five, are also commonly used in organizations. Some of them are:

• RIASEC Vocational Model or Holland Codes(Holland, 1973): Stipulates six personality types, i.e., Realistic (Doers), Investigative (Thinkers), Artistic (Creators), Social (Helpers), Enterprising (Persuaders), and Conventional (Organizers), organized into a circumplex model represented as a hexagon;
• Type A and Type B Personality Theory (Friedman, 1996): Proposes that people are either intense, hard-driving, competitive and high-achieving personalities (Type A) or relaxed, less competitive, and transcendent ones (Type B);
• HEXACO Model of Personality Structure(Ashton and Lee, 2008): Considers the Big Five dimensions plus a sixth called Honesty-Humility;
• VIA Classification of Character Strengths and Virtues (Peterson and Seligman, 2004): Based on Positive Psychology (Snyder and Lopez, 2007), it identifies an individual’s profile within a set of six dimensions (Wisdom and Knowledge, Courage, Humanity, Justice, Temperance, and Transcendence);
• Enneagram (Wiltse and Palmer, 2011): Model of human personality comprised of nine interconnected types (Reformer, Helper, Achiever, Individualist, Investigator, Loyalist, Enthusiast, Challenger, and Peacemaker) that lacks consistency in its definition and interpretation, being difficult to submit to scientific scrutiny.

All of these models have in common the fact that they seem to lack credible and unbiased empirical evidence for their psychological validity and/or their usefulness in clinical or organizational settings.

### Possible Scientific Value of Literary Fiction

Engler (2008) points out that most personality theories have a basis on a very broad set of assumptions originating from philosophy, religion, and even art. She notes that this is a natural process in the development of scientific knowledge, one that, *per se*, does not imply any lack of scientific rigor, as long as certain methods and criteria are met in the development and evaluation of such models.

Jerome Bruner proposed that the way people make sense out of life and organize their activities in daily existence is by means of a set of beliefs and practices that he referred to as “folk psychology,” a cultural phenomenon that is constructed and expressed through narratives (Bruner, 1990). Literary texts are of particularly great importance in this process as both a cause and an effect, for authors and readers simultaneously manifest and change the understanding of the self and others in themselves and in society as a whole (Bruner, 1986).

McCrae et al. (2012) argue that a literary author must provide a relatively accurate portrayal of persons and their reactions to events in order to write engaging and successful stories, something which requires from them the ability to think psychologically and to communicate their insights to others. They also quote third-party evidence, as well as results from their own empirical investigations, showing that independent assessments from different personality psychologists regarding the traits of the same literary characters (evaluated based on the traits of the NEO Inventories of the Five Factor Model) yielded similar results in the case of Goethe’s Faust, Moliere’s Alceste, and Voltaire’s Candide.

Crysel et al. (2015) took the matter one step further by evaluating the validity of a fictional system of classification in terms of identifying real-life personality traits. They considered, within the context of the extremely successful Harry Porter fantasy books, the Hogwarts school four communities or “houses” (Gryffindor, Hufflepuff, Ravenclaw, and Slytherin), that are presented as corresponding to characters’ specific traits. Their findings indicate that an individual’s inclination toward Ravenclaw was associated to measures of Need for Cognition, whereas the inclination toward Slytherin was related to measures the Dark Triad traits, both results being in accordance with the depiction of such “houses” in the stories. Though some additional expected associations were not confirmed (i.e., Gryffindor with Extraversion and Openness, as well as Hufflepuff with Need to Belong), the authors concluded that fiction can reflect real underlying personality dimensions.

All things considered, it would seem that fictional literature can be the source of coherent and realistic depictions of human personality traits, as well as provide relevant insights into the functioning of human psychology to the point of creating systems of classification with some scientific value.

### The Divergent Series

#### Origins and Psychological Inspiration

Divergent is a young-adult dystopian novel series written by the American author Veronica Roth, and later turned into a movie franchise, that was very well received by the public and critics (The Wrap, 2014; Dominus, 2011).

Roth (2011) declared that her inspirations for the plot in the Divergent trilogy included an interest in government systems that divide people into classes or castes and “an obsession with personality tests,” particularly the Meyers-Briggs Type Indicator and the Enneagram. She also was taking an introductory course in psychology at the time of writing, and was particularly impressed with exposure therapy in the treatment of phobias and the Milgram experiment on obedience to authority figures (Dominus, 2011; Roth, 2011). However, she never intended to produce or reproduce a theory or model of human personality, nor has she ever made any claims regarding the scientific validity or practical value of the concepts presented in her literary work.

#### Story Background

The story of the Divergent trilogy is set within the dystopian, isolated, post-apocalyptical city of Chicago, where order is maintained by dividing the population into five Factions, each with its own values, patterns of behavior, social functions and personality traits of its members. At the age of 16, every individual is required to, with the help of a personality test, choose to which Faction he or she wants to belong to and live with for life. Failure to live up to the standards of one’s Faction will lead to expulsion, making one “Factionless” and socially excluded, a fate considered to be “worse than death.” The plot of Divergent revolves mainly around the fact that the protagonist (“Beatrice Prior” or “Tris”) has inclinations toward Abnegation, Dauntless and Erudite all at the same time, instead of to just one of the Factions, which means facing possible persecution and death (Roth, 2013).

#### The Factions

A brief list of each Faction, its social function, and main psychological characteristics that are attributed to them, as idealized by Roth (2013) is summarized in **Table 1**.

**Table 1:** Summary of the Factions based on descriptions by Roth (2013).

Faction	Social functions	Psychological characteristics
Abnegation (The Selfless)	Government, public service and social assistance	Altruism, support of others, focus on duties and obligations, attention to details, organization, self-discipline, religiousness
Amity (The Peaceful)	Agricultural production, counseling and caretaking	Pacifism, valuing social harmony, forgiveness, desire to please, taste for pleasure and entertainment, hedonism
Candor (The Honest)	Application of the Law and trials	Frankness, honesty, energy, seeking attention and interaction with others, positive emotions, talkativeness
Dauntless (The Brave)	Defense and maintenance of order	Thrill-seeking, courage, capacity to overcome fear, competitiveness, assertiveness, importance given to physical fitness
Erudite (The Intelligent)	Teaching, research, technology, medicine and librarianship	Intelligence, curiosity, eloquence, appetite for knowledge and information, creativity, critical thinking, appreciation of art


Roth (2013) describes the five Factions in Divergent as philosophical responses to the human faults considered to be “the cause of all the evil faced by humankind.” Each Faction has its own “Manifesto” which states its belief in the overwhelming relevance of the particular vice it stands against and how they propose to overcome it. Thus, Abnegation fights selfishness with selflessness, Amity counters aggression with pacifism, Candor combats duplicity with honesty, Dauntless opposes cowardice with bravery, and Erudite attempts to defeat ignorance with knowledge (Roth, 2013). It is relevant to note that the plot seems to lead the author of the books, perhaps unwittingly, toward a more profound depiction and characterization of Abnegation, Dauntless and Erudite than of Amity and Candor.

#### The Faction Quiz

In annex to The Divergent Series Complete Collection is the Faction Quiz (Roth, 2013), which contains seven questions with five possible answers each, one for every Faction. It was designed specifically to help the reader have a feeling of his or her inclination toward each of the five Factions. The form corresponds to the allocation of seven points into five categories, so that each Faction may receive as little as zero points and as many as seven, though the more points one assigns to one Faction, the fewer are left to be assigned to another. This is a non-validated instrument created solely for the purpose of entertainment, but that can be considered as a legitimate measure of the concept of each Faction as defined by Roth (2013).

## Research Problem

Considering the notion that personality theories tend to naturally come from philosophy, religion and art (Engler, 2008), and that such sources can be seen as having the potential to tap into elements of folk psychology (Bruner, 1986, 1990), due to the need to depict credible psychological traits for fictional characters (McCrae et al., 2012), it makes sense to investigate the possible scientific value of compelling fiction in terms of providing a basis or inspiration for useful models (McCrae et al., 2012; Crysel et al., 2015).

The Factions system in the Divergent series (Roth, 2013) might very well serve as a source of concepts for a new and improved scientific understanding of human personality. The basic idea is that humankind has five fundamental flaws which threaten its collective existence: selfishness, aggression, duplicity, cowardice and ignorance. To counter them, there are five corresponding broad sets of values, behaviors and traits from which five specific forms of functioning in society emerge. From the descriptions in **Table 1**, one can argue that:

• Abnegation is characterized as being the rigorous adherence to an ethos of altruism, steering one’s actions toward effectively helping others, generally with a religious attitude. This implies core values regarding being supportive of the needs of fellow human beings even in detriment of one’s own needs, something tied to cultural traditions that focus on the community, emphasize discipline, favor social stability and manifest themselves in concrete practices. A substantial level of self-control is required for that, implying in strong mechanisms of emotional regulation and a high level of Conscientiousness. This would favor a lower level of Neuroticism (or a higher level of Stability). On the other hand, the introjection and internalization of such values would be expressed as a higher level of Agreeableness.
• Amity is simplistically described as being essentially oriented toward social harmony, individual happiness and hedonism. As such, its is associated to values regarding the satisfaction of personal desires and interacting well with others, as well as to the time spent on rest and relaxation.
• Candor is basically depicted as being frank and outgoing, seeking the attention of others. This essentially corresponds to Extroversion in the Big Five model. It is perhaps the Faction that was least elaborated.
• Dauntless is conceived as being assertive, competitive and thrill-seeking, with a great attention to physical prowess and feats. Therefore, it is expected to be associated to valuing status and influence, sexual activity, and being an “adrenaline junkie.” It is also expected to be associated to dedicating time toward attaining physical fitness.
• Erudite is defined as an inclination toward all things intellectual, scientific and technological, which suggests that it encompasses traits regarding cognitive ability and skills, educational attainment, and the relationship with technology and the Digital Revolution. It is to be expected that such traits be related to personal values regarding the acquisition of knowledge and the appreciation of art, as well as pertaining to the development and use of one’s potentials in that regard. This would translate into dedicating more time to professional work, which would tend to be mental in nature, as well as to “extracurricular” or “dilettante” interests. In the Big Five Model, these characteristics are within the realm of Openness to Experience.

It is, therefore, possible that the Factions constitute a classification of traits that can be regarded as an “anatomy” of human personality that categorizes cognition, relationship with knowledge and technology, emotional regulation, values, and the allocation of time, as well as to the Big Five dimensions.

It should be noted that the best available models of personality are frameworks where one considers multiple dimensions of personality that are present, to varying degrees, within each individual, rather than a mere classification of people into just one of multiple types (Goldberg, 1980, 1981; Digman, 1990; Pittenger, 1993a,b; Gardner and Martinko, 1996; Coffield et al., 2004). This is in agreement with the main plot of the Divergent series, where the idea of classifying every member of a population into one of five mutually exclusive Factions ultimately fails (Roth, 2013). Thus, in the present paper the choice was made to study the accuracy of the Factions system in terms of constituting relatively independent overarching dimensions of personality rather than as types *per se*.

It is also worth observing that the design of the Faction Quiz allows for only seven possible items per Faction, plus a scoring system that creates artificial negative correlations between Factions, both things making it difficult to perform a meaningful traditional factor analysis of the relational structure or to calculate reliability score through a Cronbach Alpha (Cronbach, 1951; Kline, 2000; Voss et al., 2000; Swailes and McIntyre-Bhatty, 2002). This implies a lower level of precision (i.e., a higher level of dispersion) in the evaluation of the dimensions assessed, even though there are precedents for valid instruments using such format (e.g., Belbin, 1990). However, the Faction Quiz has the virtue of being a legitimate reflection of the classification introduced by Roth (2013). Furthermore, if the constructs appraised are robust, their relationship to other psychological variables might be strong enough to emerge even if measured under a relatively high margin of error. Thus, it is reasonable to attempt to use such an instrument in preliminary evaluations of validity and, in finding encouraging initial results, later produce an improved version of the test.

## Study Goals

The present study has two aims. The first is to scientifically investigate the accuracy of the Factions, as described in the Divergent trilogy and measured by the Faction Quiz (Roth, 2013), in terms of describing specific combinations of cognition, relationship with knowledge and technology, emotional regulation, values, and the allocation of time that may be considered as constituting overarching personality traits. The second is to assess the practical usefulness of these Faction dimensions for the understanding of social life, particularly in the context of work life choices and experiences in organizations.

## Materials and Methods

### Participants

A total of 217 individuals from the Metropolitan Area of Recife, Pernambuco, Brazil, being 101 men (46.5%) and 116 women (53.5%), with a mean age of 35.7 years (*SD* = 14.52). Roughly 40.6% had fundamental level of schooling, 17.5% high school, 20.7% a higher education degree, and 21.1% a graduate degree.

### Materials

The following instruments were chosen to measure sociodemographics, cognition, relationship with knowledge and technology, emotional regulation, values, allocation of time, the Big Five dimensions, and work life, most selected due to their practicality in terms of quickly and easily assessing the desired variables, as well as because of validity:

• Sociodemographics and Work Life Form: An especially prepared form containing questions regarding sex, date of birth, marital status, income, religion, level of education, field of education, type and segment of occupation, position at work, job satisfaction, satisfaction with relationships at work, and personal time dedicated to sports/physical exercise, work, sleep, and other activities;
• Hyperculturality Form (Souza et al., 2012): A form containing questions regarding one’s relationship with Information and Communications Technologies (ICT) and the sociocultural elements created around them, from which one can calculate the following measures:
  ○ Hypercultural Index: The level of internalization of the thinking and acting of the Digital Age, which includes mastery of the use of ICT, scientific and mathematical thinking, abstract thought, multitasking, and fragmentation of knowledge;
  ○ Experience With ICT: The number of years passed since one began to use computers regularly;
  ○ Digital Precociousness: The inverse of the age in which one began to use computers regularly.
• Mini IQ Test (Souza et al., 2010): A very short intelligence test containing a total of five questions involving mental reversal of images, use of geometric knowledge and visualization, word analogies, and mathematics;
• General Knowledge Test (Souza et al., 2010): A short test containing 10 simple “true” or “false” questions in high school mathematics, physics, chemistry, biology, history, geography, Portuguese, and English;
• Basic Values Questionnaire (Gouveia et al., 2013): An instrument designed to measure basic human values according to the Functionalist Theory, comprising 18 specific values or markers to be self-appraised on a scale that varies from 1 (totally unimportant) to 7 (highest importance), such values being:
  ○ Affectivity: Have a deep and lasting affective relationship; have somebody to share one’s successes and failures;
  ○ Beauty: Be capable of appreciating the best of art, music and literature; go to museums or expositions where one can see beautiful things;
  ○ Belonging: Get along with one’s neighbors on a daily basis; being part of a some social, sporting or other type of group;
  ○ Emotion: Enjoy challenging danger; seek adventure;
  ○ Health: Concern with one self’s health even before one becomes sick; not being physically or mentally ill;
  ○ Knowledge: Search for updated news regarding little-known subjects; try to discover new things about the World;
  ○ Maturity: Feel that one has attained one’s objectives in life; develop all of one’s capacities;
  ○ Obedience: Fulfill one’s day-to-day duties and obligations; respect one’s parents, superiors and elders;
  ○ Personal Stability: Be certain that tomorrow one will have all one has today; have an organized and planned life;
  ○ Pleasure: Enjoy life; satisfy all one’s desires;
  ○ Power: Have power to influence others and control decisions; to be the head of a team;
  ○ Prestige: Know that many people know and admire you; in one’s old age, receive a tribute for one’s contributions;
  ○ Religiosity: Believe in God as the savior or humanity; fulfill God’s will;
  ○ Sexuality: Have sexual relations; obtain sexual pleasure;
  ○ Social Support: Obtain help when one needs it; feel that one is not alone in the World;
  ○ Success: Achieve what one proposes oneself to obtain; be efficient in all one does;
  ○ Survival: Have food, water, and be able to sleep well every day; live in a place with an abundance of food;
  ○ Tradition: Follow the social norms of one’s country; respect the traditions of one’s society.
• Ten Item Personality Inventory (Gosling et al., 2003): A standardized brief measure of the Big Five dimensions (Openness, Conscientiousness, Extraversion, Agreeableness, and Stability), that was structurally validated in Brazil by Souza et al. (2013);
• Emotional Regulation Questionnaire (Gross and John, 2003): A standardized measure of emotional regulation, validated in Brazil by Boian et al. (2009), that yields two measures.
  ○ Emotional Suppression: Tendency to regulate emotions by means of controlling their expression;
  ○ Cognitive Reappraisal: Regulation of emotions by means of the reinterpretation of the underlying situation.
• Faction Quiz (Roth, 2013): Questionnaire with seven questions designed to measure one’s inclination toward each of the Divergent series Factions (Abnegation, Amity, Candor, Dauntless and Erudite), generating a 0–7 score for each based on the answers, but also allowing the calculation of a “Divergence” score based on the inverse of the mean deviation of the scores given to each faction.

### Procedures

A total of 16 students from the Graduate Program in Business Administration of the Federal University of Pernambuco, approached the subjects in the streets of the Metropolitan Area of Recife, Pernambuco, Brazil, explained the nature and purpose of the investigation, invited them to participate, and applied the instruments to those who accepted. The application was done in various locations according to convenience, but always within the setting of a quiet room with a closed door and a place to sit and write. The experimenters were each instructed to collect anonymous data on 16 subjects, to be equally divided into: (a) men and women, (b) those aged 30+ years and those younger, and (c) those with intermediate level of education or more and those with less schooling. A total of 256 subjects were obtained, leading to a final dataset of 217 after 39 were discarded due to incomplete or incorrect records.

### Analysis

Social and human phenomena are typically extremely complex due to the large amount of variables involved and the intricate covariance between them. Usually, the relationship between variables A and B seems to depend on the relationship between each of them and other variables, which interact in a similar fashion with yet other variables, and so forth. The major challenge is to find overarching patterns in a broad and convoluted set of observations to inspire and/or put to test scientific models regarding mechanisms and processes. The prominent Israeli–American mathematician, sociologist and psychologist Louis Guttman developed a theoretical-analytical framework, called Facet Theory, which addresses precisely such issues. It allows for a spatial representation of the relational structure of a large number of variables simultaneously. This is achieved through the concept of a “mapping sentence,” which permits the identification of clusters, latent variables, and/or constructs by means of a simple partitioning of the geometric representation of a multivariate space. Such space can consist of literally any kind of data, form of relationship, and measure of association, visually expressing all the relationships, including covariances, in a very intuitive way (Guttman and Greenbaum, 1998; Levy, 2005). It has significant advantages to Factor Analysis and Cluster Analysis in terms of weaker assumptions, more precise expression of associations, and the better integration of the findings to a theoretical framework (Guttman, 1992; Maslovaty et al., 2001; Borg and Groenen, 2005).

The current investigation included 68 variables of different types (psychological, sociodemographic, organizational), measured in different scales (dichotomous, ordinal, ratio), and with both linear and non-linear relationships between them. Additionally, the large number of variables measured introduces numerous possible confounding factors and mediation effects that render simple bivariate analysis virtually meaningless.

Considering all the above, plus the fact that the groupings of variables that were derived from the descriptions of each Faction in **Table 1** constitute a veritable mapping sentence, the decision was made to use SSA and Facet Theory as the approach to deal with both the scope and the complexity of the data involved, with Stasoft Statistica 10 as the software tool.

For each SSA, the solution chosen was the one with the lowest number of dimensions that still obtained levels of Alienation and Stress below.20, considered reasonable for the relatively large number of variables involved. Dimensions 1 and 2 were privileged because this view of the multidimensional space approximates very well what is to be found in a two-dimensional solution, which is simpler to interpret. Multiple projections are possible, but one is not required to explore all them to validate the interpretation of one (Guttman and Greenbaum, 1998; Borg and Groenen, 2005; Levy, 2005).

## Results

### Factions vs. Psychological Variables

**FIGURE 1:**
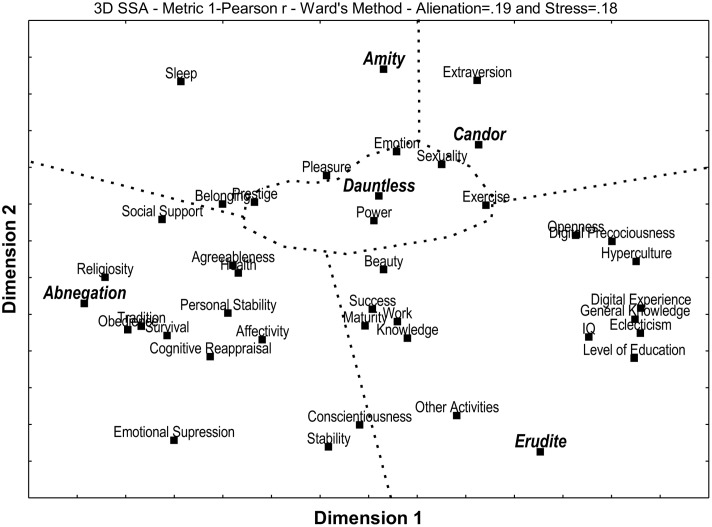
Smallest Space Analysis (SSA) for the five Factions and the cultural-psychological variables.

**Figure 1** shows an SSA displaying the multiple relationships between the scores obtained for each Faction on the Faction Quiz and the measures of: the Big Five personality traits, basic human values, emotional regulation, IQ, hyperculturality, general knowledge, level of education, eclecticism (no. of different fields in which one has some level of formal education), and use of time (for exercise, sleep, work, and other activities).

One can partition the SSA space in **Figure 1** into five contiguous regions, one for each Faction and its specific set of cultural and psychological variables as indicated by Roth (2013). The resulting structure is a radix (combination of polar and modular structures) with Dauntless at the center and the remaining Factions surrounding it.

Abnegation was in the same partition as the basic values of Obedience, Tradition, Religiosity, Survival, Social Support, Personal Stability, Health and Affectivity. It was also linked to both aspects of emotional regulation that were measured, namely, Cognitive Reappraisal and Emotional Suppression, as well as to the personality dimensions of Agreeableness, Conscientiousness and Stability.

Amity was linked to the values of Belonging and Pleasure, along with the time dedicated to sleep.

Candor was grouped only with the Big Five dimension of Extraversion.

Dauntless was related to the values of Emotion, Power, Prestige and Sexuality, and also to the time spent on physical exercise or sports.

Erudite was in the same partition as the cognitive variables of IQ, General Knowledge, as well as Digital Age, Digital Precociousness and Hyperculture, along with level of education and Eclecticism. It was also linked to the Big Five dimension of Openness to Experience, along with the values of Knowledge, Maturity, Success and Beauty. There was a link to the amount of time spent working, plus to the time spent on activities other than work, exercise or sleep.

### Factions vs. Work Choices

**FIGURE 2:**
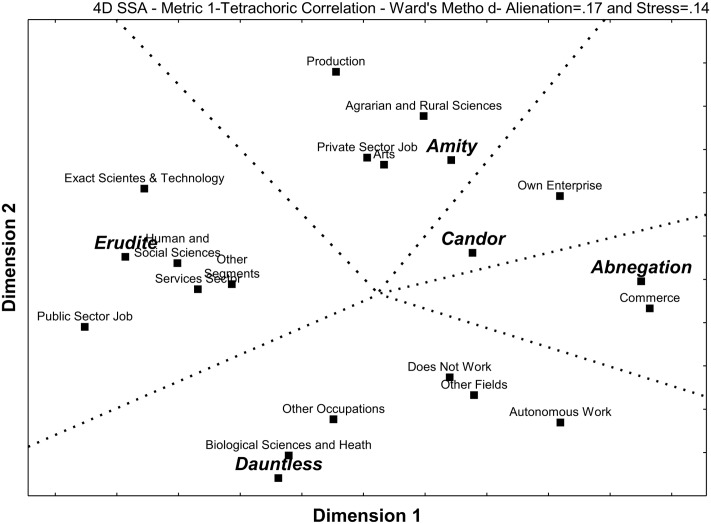
Smallest Space Analysis for the five Factions and work choices.

**Figure 2** shows an SSA displaying the multiple relationships between the scores obtained for each Faction on the Faction Quiz and the field of education, type of occupation, and segment of work. Given that most of these variables are dichotomous, here the scores for each Faction were dichotomized at the median so as to establish a common denominator.

Here one can partition the SSA space in **Figure 2** into five contiguous regions, one for each Faction and its specific set of work choices. The resulting structure is a polar pattern.

Abnegation was associated to working in commerce. Amity was linked to having a background in Agrarian and Rural Sciences and/or in Arts, as well as with having a Private Sector Job or one in Production. Candor was related to having one’s Own Enterprise. Dauntless was linked to a background in Biological Sciences and Health, and/or to one in a field other than Exact Sciences and Technology, Biological Sciences and Health, Human and Social Sciences, Agrarian and Rural Sciences or Art; it was also associated to Autonomous Work, occupations other than Private Sector Job, Public Sector job or Autonomous Work, and to not working. Erudite was associated to a background in Exact Sciences and Technology, but also to one in Human and Social Sciences, as well as to working in the Services Sector and likewise to working in sectors other than Services, Commerce or Production.

### Factions vs. Work Life

**FIGURE 3:**
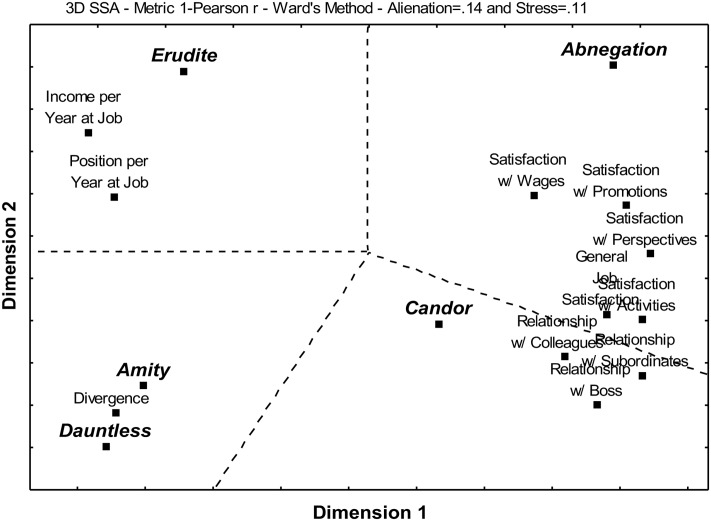
Smallest Space Analysis for the five Factions and work life experiences.

**Figure 3** shows an SSA displaying the multiple relationships between the scores obtained for each Faction on the Faction Quiz and income per year at job, hierarchical position at work per year at job, job satisfaction, and satisfaction with relationships at work.

One can partition the SSA space in **Figure 3** into five contiguous regions, one for each Faction and its specific set of work life experiences. The resulting structure is a polar pattern.

Amity and Dauntless were associated with each other and to Divergence, but not to any particular aspect of work life experiences. Abnegation was linked to General Job Satisfaction, Satisfaction w/ Activities, Satisfaction w/ Wages, Satisfaction w/ Promotions, and Satisfaction w/ Perspectives. Candor was related to Relationship w/ Boss, Relationship w/ Colleagues, and Relationship w/ Subordinates. Erudite was linked to Income per Year at Job and Position per Year at Job.

## Discussion

### The Factions in Divergent and Their Psychological Associations

#### The Psychology of Abnegation

**Figure 1** shows that the basic human values associated to the scores in Abnegation were Tradition and Obedience (a tendency for self-discipline and conformity), Social Support and Affectivity (a need for interpersonal security), Survival, Health and Personal Stability (material pragmatism), and Religiosity (belief in God). This Faction was also related to the two types of emotional regulation, that is to say, Cognitive Reappraisal and Emotional Suppression (both indicative of self-control), and to the Big Five personality dimensions of Conscientiousness (discipline, organization and a focus on duty), Agreeableness (concern for others) and Stability (impulse control and ability to deal with negative feelings).

#### The Psychology of Amity

**Figure 1** shows that the Amity scores were related to the human values of Belonging (need to be a part of a group and to get along well with others) and Pleasure (hedonism). There was also an association with the time spent sleeping (rest).

#### The Psychology of Candor

In **Figure 1**, Candor scores were shown to be associated to the Big Five dimension of Extroversion, indicating assertiveness, talkativeness, frankness, and a tendency toward seeking company and attention.

#### The Psychology of Dauntless

**Figure 1** shows Dauntless as linked to the moral values of Power (aspiration to leadership and desire for influence), Prestige (seeks to impress others), emotion (search for excitement) and Sexuality (interest in sex). It was also associated to the time spent on physical exercises and the practice of sports (fitness and athleticism).

#### The Psychology of Erudite

In **Figure 1**, Erudite was shown to be associated to IQ, Level of Education, Eclecticism, Digital Experience, Digital Precociousness, and Hyperculture (cognitive ability and skills). It was related to the basic values of Knowledge (maintain one’s self updated and discovering things about the World), Maturity (attaining one’s goals and developing one’s full capacity), and Beauty (appreciation of the best of art, music and literature in museums or expositions). There were also links to the Big Five dimension of Openness to Experience (intellectualism, preference for novelty and variety, and appreciation of art). This Faction was likewise associated to the amount of time spent working (perhaps skilled, technical or otherwise intellectual in nature), plus to the time spent on activities other than work, exercise or sleep (possibly studying, creating and/or engaging in intellectual pursuits).

### Validation of Faction Psychology and Potential Additional Insights

The findings of the present study strongly suggest the existence of a remarkable similarity between the psychological profile of the Factions as conceived by Roth (2013) and the actual associations that were observed between the results of the Faction Quiz and the various psychological measurements that were made. For each Faction, one can find a statistical link either to variables that are a direct expression of that Faction’s stated characteristics and behaviors, or to psychological mechanisms that clearly explain them. In fact, the Factions were more scattered through the multidimensional space of **Figure 1** than the Big Five dimensions, indicating that former have greater explanatory power than the relatively “clustered” latter.

Furthermore, the radix that was found in **Figure 1** suggests that Amity and Erudite are in direct opposition to each other, as are Abnegation and Candor, with Dauntless playing a prominent role at the center, i.e., positively associated to all of the other Factions. Further studies to determine whether such a structure is circumstantial to the present investigation, as in a characteristic of the particular sample studied, or if it reflects a more generalized characteristic of human personality. In the latter case, it would be necessary to theorize as to the cause, for such a structure is not to be expected directly from Roth (2013), save for the intriguing fact that the plot focuses on Dauntless as the chosen Faction of the main character (which would put it at the center) and that Abnegation and Erudite are the most complex and detailed Factions (making them occupy the greatest portions of the diagram).

### Factions and Work

#### Factions and Social Functions in the Fictional Divergent Universe

In the Divergent trilogy, Roth (2013) assigns specific roles to each of the Factions in the fictional, dystopian and post-apocalyptical city of Chicago (**Table 1**). It is important to be aware that such designations are made within the context of a simplistic and unrealistic society with equally simplistic and unrealistic organization, production relations, economy, and culture. In a more lifelike scenario (i.e., a far more complex one) there might not be the same links between social functions and Faction personality dimensions as expressed in the literary work. Nevertheless, interesting results in that regard were found in the present study.

#### Abnegation and Work

Abnegation was associated to working in Commerce (**Figure 2**) and to the satisfaction with the various aspects of work (**Figure 3**), an explanation for which might be found considering the psychological findings regarding this Faction (**Figure 1**).

Psychologically, Abnegation was found to be related to material pragmatism, but also to self-control, discipline and organization, all of which can favor the objective and methodic work commonly found in the jobs in commerce. Besides, this type of activity involves a process of negotiation through which one, to some extent, concedes to the needs of others, this being done within the context of a certain degree of trust between the parties, obedience to common rules, and the support from social mechanism of regulation, all of this resonating with both this Faction’s sense of altruism and its need for interpersonal security. On the other hand, the sense of duty and conformity, along with the aforementioned self-control, can lower one’s expectations regarding the reward from professional life, or at least one’s expression of them, favoring a stronger manifestation of satisfaction at work.

#### Amity and Work

Amity was linked to having a background in Agrarian and Rural Sciences and/or in Arts, working in Production, and having a Private Sector Job (**Figure 2**), but not with any specific aspect of work life such as satisfaction, relationships or success (**Figure 3**). This is in keeping not only with the psychological profile associated to the Faction (**Figure 1**), but also to its role in the fictional Divergent universe (Roth, 2013).

Amity is explicitly presented by Roth (2013) as the Faction in charge of agricultural production, which is in perfect agreement with the observed tendency for a background in agriculture-related fields and for working in the productive sector. Its members are also described as engaging themselves in art and entertainment, which is in full concordance not only with the empirical observations of the present study regarding a background in arts and work in the private sector, but also with the psychological findings of hedonism.

The lack of association with professional satisfaction, relationships or success can be seen as the consequence of the fact that Amity is not a human dimension that is particularly conducive to great accomplishments in most workplaces, but rather one that is associated to pleasure, conviviality and rest, which, in many cases, may be seen as the opposite of work.

#### Candor and Work

Candor was associated to being an entrepreneur (**Figure 2**) and to the quality of the relationship with co-workers (**Figure 3**). This can be considered as consistent with the psychological association found for this Faction (**Figure 1**).

The link found between Candor and Extraversion indicates that this Faction is related to being expansive, energetic and assertive, that is, to wanting to be the focus of attention of others and even to leadership. This may lead to entrepreneurism as a way of occupying center stage and/or for an individual with a high level of this trait to avoid possible conflicts between his or her intense personality and the traditional hierarchical structure of a typical job. The orientation toward social interaction, along with the trust that comes from frankness, can explain the tendency for a good relationship with co-workers.

#### Dauntless and Work

Dauntless was related to a background in Biological Sciences and Health, and/or to one in a field other than Exact Sciences and Technology, Biological Sciences and Health, Human and Social Sciences, Agrarian and Rural Sciences or Art; it was also associated to Autonomous Work, occupations other than Private Sector Job, Public Sector job or Autonomous Work, and to not working (**Figure 2**), but there was no link to professional satisfaction, relationships or success (**Figure 3**). Such a pattern can be considered as having some relationship to the previously shown psychological findings (**Figure 1**).

The association between Dauntless and the value given to influence and status might explain the decision to take up a prestigious field such as medicine (part of Biological Sciences and Health), whereas the search for danger and excitement, as well as that for physically demanding activities, might drive one for careers in the police or the military (which are not part of the traditional fields of knowledge). Taken to higher level, the thrill seeking and physicality could drive one to more “daredevil” (and, therefore, also less traditional) jobs such as being a professional athlete, mountain climber, stuntman, and so forth, many of which are performed by autonomous professionals. On the other hand, these same characteristics could, under negative circumstances, lead to criminal behavior or prevent one from keeping a job.

#### Erudite and Work

Erudite was linked to having a background in Human and Social Sciences and/or in Exact Sciences and Technology, working in the Services Segment or in segments other than Services, Commerce and Production, and having a Public Sector Job (**Figure 2**). There was also a relationship with career success at work, as measured by Income per Year at Work and Position per Year at Work (**Figure 3**).

Roth (2013) puts Erudite in charge of all things intellectual, namely, teaching, research, development of technology, medicine and librarianship (**Table 1**), which is in full agreement with the associations of this Faction with a greater depth and breadth of knowledge, a higher level of overall cognitive ability, better insertion into the Information Age, and a constant desire to learn (**Figure 1**). This is also consistent with having a public job, given that, in Brazil, the best universities and research institutes tend to be in the public sector, with working in services having a likewise relationship with being a professor and/or researcher. Furthermore, it resonates with having a background in science, technology, engineering and mathematics, as well as in psychology, sociology, communication and history, as stated in the Faction’s fictional Manifesto (Roth, 2013). It is possible that the finding of a link with working in a sector other than Services, Commerce and Production has to do with being a consultant, which is another professional activity related to knowledge.

The finding that Erudite is clearly associated to job success in terms of climbing positions and pay grades in less time can be explained by the socioeconomic factors such as the strong association between education and income and the emergence of the Knowledge Age, Information Society and Knowledge Economy.

## Conclusion

The present paper aimed to investigate whether the personality dimensions expressed in the fictional Factions of the Divergent series (Roth, 2013) could constitute a sound, functional and useful psychological framework. For this purpose, a relatively large number of diverse subjects from Recife, Pernambuco, Brazil, were submitted to the Faction Quiz and various instruments that measured Big Five personality traits, basic human values, emotional regulation, cognition, relationship with ICT, sociodemographics, use of time, work choices, and work life experiences.

Multivariate analysis of the dataset using SSA and Facet Theory obtained results that strongly indicate that the Factions, as psychological dimensions associated to the various psychological variables in ways that are very much in agreement with the definitions, descriptions and plot from Roth (2013), perhaps more meaningful than in the case of the Big Five, with regards to associating to a broader scope of variables. Furthermore, they showed specific links to work choices and experiences that are quite consistent with their psychological associations and, to a limited extent, to their social roles in the Divergent universe (Roth, 2013).

It is concluded that the five Factions conceived by Roth (2013) appear to constitute an original set of constructs that not only synthetize various motivational, emotional, cognitive, and behavioral variables in a coherent and meaningful way, but also seem to be of practical value in the understanding of how individuals relate to their work. This suggests that the basic premises underlying the origin and nature of the Factions, that is, as psychosocial responses to the existential threats of selfishness, aggression, duplicity, cowardice and ignorance, might be a valid basis for the development of a new approach to human personality, one with concrete implications for people management in organizations, among other possibilities.

Besides replicating the present study within the context a larger, broader, and more diversified sample, as well as including a wider range of psychological variables, future investigations on the subject should focus on the development of an improved version of the Faction Quiz, capable of more precise and reliable measurements of the dimensions of Abnegation, Amity, Candor, Dauntless and Erudite. It is of even greater importance to create theories that might explain the causes and functioning of the dimensions in question.

## Ethics Statement

As established by the ethical guidelines for scientific research with human subjects in Article 1, Subsection V, of Resolution no. 510 from the Brazilian National Council on Health, the present study was exempt from registration or evaluation from the country’s Council of Ethics in Research and National Council of Ethics in Research due to the fact that no identification of subjects was registered or even asked for, no experimental intervention was done on the participants that might generate any risks above those of daily life, and absolutely no form of diagnosis or counseling was offered either as a consequence of the responses or any other basis. In accordance to international principles regarding research ethics, the participation in the present study was fully informed and strictly voluntary.

## Author Contributions

Both authors (BdS and AR) equally contributed to all the following issues of the research: conception and design of the work; acquisition, analysis, and interpretation of data; drafting the manuscript and critically revising it; final approval of the version to be published; agreed to be accountable for all aspects of the work in ensuring that questions related to the accuracy or integrity of any part of the work are appropriately investigated and resolved.

## Conflict of Interest Statement

The authors declare that the research was conducted in the absence of any commercial or financial relationships that could be construed as a potential conflict of interest.
